# A COVID-19 Rumor Dataset

**DOI:** 10.3389/fpsyg.2021.644801

**Published:** 2021-05-31

**Authors:** Mingxi Cheng, Songli Wang, Xiaofeng Yan, Tianqi Yang, Wenshuo Wang, Zehao Huang, Xiongye Xiao, Shahin Nazarian, Paul Bogdan

**Affiliations:** ^1^Ming Hsieh Department of Electrical and Computer Engineering, University of Southern California, Los Angeles, CA, United States; ^2^School of Computer Science, Fudan University, Shanghai, China; ^3^Department of Automation, Tsinghua University, Beijing, China; ^4^UM-SJTU Joint Institute, Shanghai Jiao Tong University, Shanghai, China; ^5^Department of Automation, Beihang University, Beijing, China

**Keywords:** misinformation, rumor, COVID-19, rumor classification, sentiment analysis, deep learning, fake news detection

## 1. Introduction and Summary

The outbreak of the coronavirus disease (COVID-19) has caused widespread concerns among the public and has profoundly impacted social opinions since December 2019 (Nelson et al., 2020). Besides the disease itself, the pressure caused by the pandemic and the fear of disease spreading have become a psychological burden (Abdoli, 2020). Authors in (Luo et al., 2020) showed that patients with pre-existing conditions and COVID-19 infection are experiencing psychological distress, anxiety, and depression. The ongoing pandemic has also aroused heated discussion on the Internet (Cinelli et al., 2020). In response to the infodemic, scientists rise and fight against rumors, conspiracy, misinformation, fake news, and disinformation (Gallotti et al., 2020; Jolley and Paterson, 2020; Butcher, 2021; Cheng et al., 2021b). In this, all sorts of information are disseminated in the form of news reports, tweets, etc. (Kouzy et al., 2020). Topics of public discussion vary over time as the virus spreads rapidly, while real and fake information is mixed, leading to increasing confusion in some communities. Rumors are defined as statements or reports currently without known veracity concerning their truthfulness, (Friggeri et al., 2014) which spread misinformation/disinformation (deliberately misleading information) and cause panic, hatred, and discrimination (Depoux et al., 2020). Along with the rapid dissemination of misinformation, researchers discover a massive growth in fact-checks about COVID-19, e.g., the number of English-language fact-checks increased more than 900% from January to March 2020 (Brennen et al., 2020). Fact-checking websites such as the FactCheck.org (Brooks, 2020) and Poynter.org (Neil Brown, 2020) are the primary sources of current COVID-19 misinformation/rumor data.

**Figure 1:**
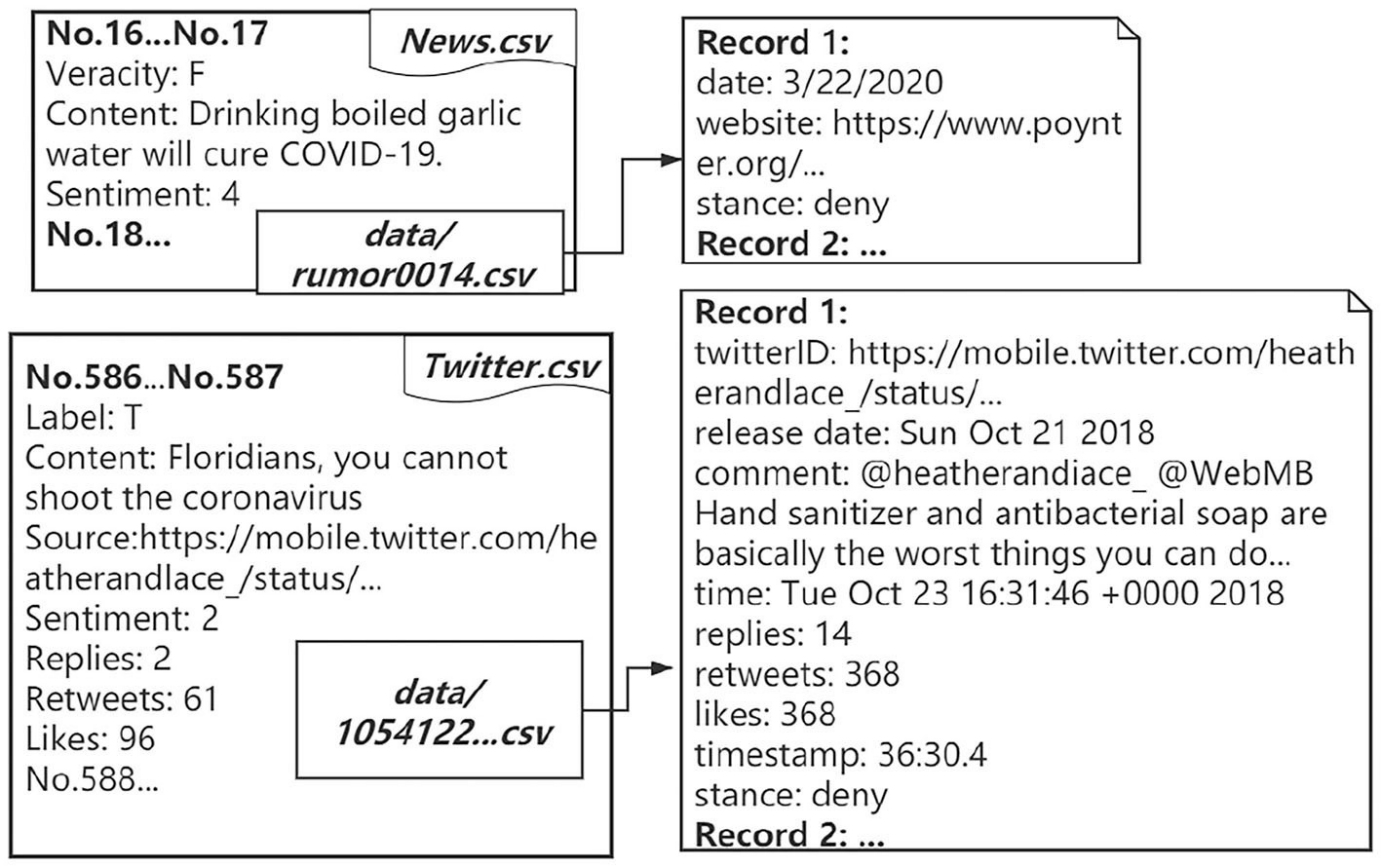
Examples of data structure.

COVID-19 rumor datasets have been collected, e.g., the COVID-19-TweetIDs dataset contains an ongoing collection of tweets IDs associated with the COVID-19 tweets (Chen et al., 2020); the COVID-19-Arabic-Tweets-Dataset contains a collection of Arabic tweets IDs related to the COVID-19 (Alqurashi et al., 2020); the CoAID (Covid-19 healthcare misinformation dataset) is a diverse COVID-19 healthcare misinformation dataset, including fake news on websites and social platforms, along with users' social engagement with such news (Cui and Lee, 2020). Besides the veracity labels and sources provided in the above-mentioned fact-checking platforms, other meta-information, such as sentiment and stance, is missing in most datasets. Therefore, COVID-19 rumor datasets for the study of sentiment analysis and other rumor classification tasks, including stance verification of COVID-19 rumors, are still in great need. In this work, we collect COVID-19 rumors and manually label 6, 834 data (4, 129 rumors from news and 2, 705 rumors from tweets) with sentiment and stance labels. The veracity status of the rumors is collected from fact-checking websites. Moreover, we include 32, 750 reposts for news rumors and 34, 847 retweets for Twitter rumors and manually label the stance. Examples of our data structure and statistics are shown in Figure 1 and Table 1. Besides, we provide analyses of our dataset, including the statistical analysis of the rumor dissemination phenomena and classification results on a deep learning-based rumor classifier.

**Table 1:** Summary of data records.

**Dataset**	**Records**	**Month**	**Replies**	**Frequency (%)**
News	4,129	Jan.	3,164	9.6
		Feb.	5,413	16.5
		Mar.	15,310	46.7
		Apr.	1,895	5.8
		Others	6,968	21.4
Tweets	2,705	Jan.	12,702	35.1
		Feb.	9,350	25.8
		Mar.	12,646	34.9
		Apr.	1,386	3.8
		Others	146	0.4

## 2. Methods

We collect rumors from a wide range of sources and refer to various authoritative fact-checking platforms (for their comprehensive analysis), e.g., *poynter.org, factcheck.org*. We also collect online discussions from Twitter, which contained real-time discussions with specific tags. To gather information from Twitter, we focus on several relevant tags and official accounts (e.g., ABC News, Reuters, CNN, and BBC News) to trace the updates of the hot topics. We record the rumor sentences in our dataset and enrich the dataset with more details, such as the source website, date of publication, veracity, sentiment, and stance. We also include reposts or retweets of the rumors and provide their stance labels. We separate our collected rumors into two datasets based on their source: (i) a news dataset, containing rumors collected from news websites, and (ii) a Twitter dataset, containing rumors collected from Twitter.

### 2.1. Data Collection

**Figure 2:**
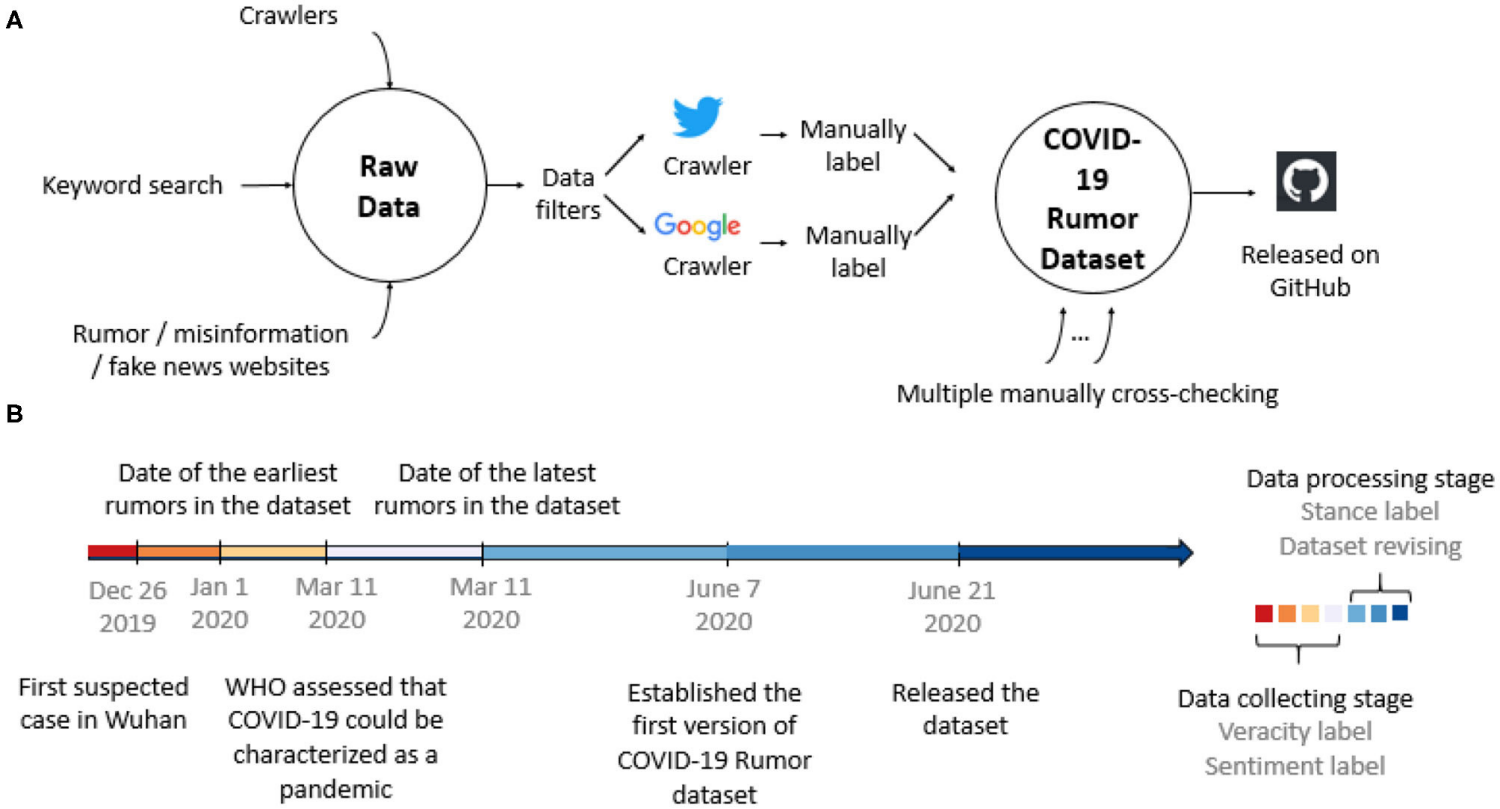
**(A)** Dataset collection, labeling, and post-process flowchart. **(B)** Timeline of dataset establishment and major events in the outbreak of the COVID-19 pandemic.

We have developed web crawlers to extract data automatically from the Google browser and Twitter. The codes are all available on our GitHub repository. The data collection process and timeline are illustrated in Figure 2.

#### 2.1.1. Tweets Collecting Method

We collect tweets with COVID-19 related tags, such as *COVID-19, coronavirus, COVID*, and store them in .*csv* files in the format of *fakeID, release_date*, and *full_text*. Duplicated tweets are dropped. Then, the sentiment of each rumor is labeled through careful analysis of the emotion of the rumor content and context. Moreover, we fetch metadata of each tweet, including reply/retweet comment content, reply number, retweet number, like number, and publish date. These metadata are saved in individual files named by the tweets' fakeIDs. Then we manually label the stance of the replies or retweet comments.

#### 2.1.2. News Collecting Method

We use the *mitmproxy* (Cortesi et al., 2020), an open-source interactive HTTPS proxy, to collect detailed information related to selected news reports from Google browser. Unlike the Twitter crawler, the Google crawler mainly catches data from the search result page and queries the results' absolute and relative paths according to the *URL* and date. We save the news rumors in a *news.csv* file, and the repost records are saved in *rumorID.csv*'s. Each rumor record has a veracity label and its content, and each repost record has a repost date, repost website, and stance label. It is worth noting that not all source website contains date information.

## 3. Data Records

The full data are freely available on GitHub. All records are stored in .*csv* files. Utf-8 encoding is recommended for the best display. We build two datasets to store the rumors from news and Twitter, respectively. Metadata is provided for further research use and analysis; meanings of the labels are demonstrated in Table 2.

**Table 2:** Demonstration of labels in the dataset.

**Term**	**Label**	**Explanation and examples**
Veracity	True (T)	The content is logical and describing the facts, e.g., “Wuhan has been quarantined.”
	False (F)	The content is made up, or contains false information, e.g., “Drinking bleach can cure coronavirus.”
	Unverified (U)	The authenticity or truthfulness of the statement is hard to judge at the time of labeling.
Stance	Support	Positive attitudes about the content, e.g., “I think the statement is right.”
	Deny	Denying attitudes about the content, e.g., “Are you kidding? This is wrong!”
	Comment	No obvious stance, e.g., “This message is interesting.”
	Query	Doubting the validity of news/tweets, e.g., “Is that true?” or “Can you prove?”
Sentiment	Very Negative (0)	The content has a strong pessimism.
	Negative (1)	The emotion is pessimistic but weaker than “very negative”
	Neutral (2)	The comment/report is in a plain and narrative tone.
	Positive (3)	The content reflects positive emotions or aims, such as news providing tips to fight the virus.
	Very Positive (4)	Cheerful news such as progress in the research, massive donations or breakthroughs in the vaccine.

### 3.1. News Dataset

**Figure 3:**
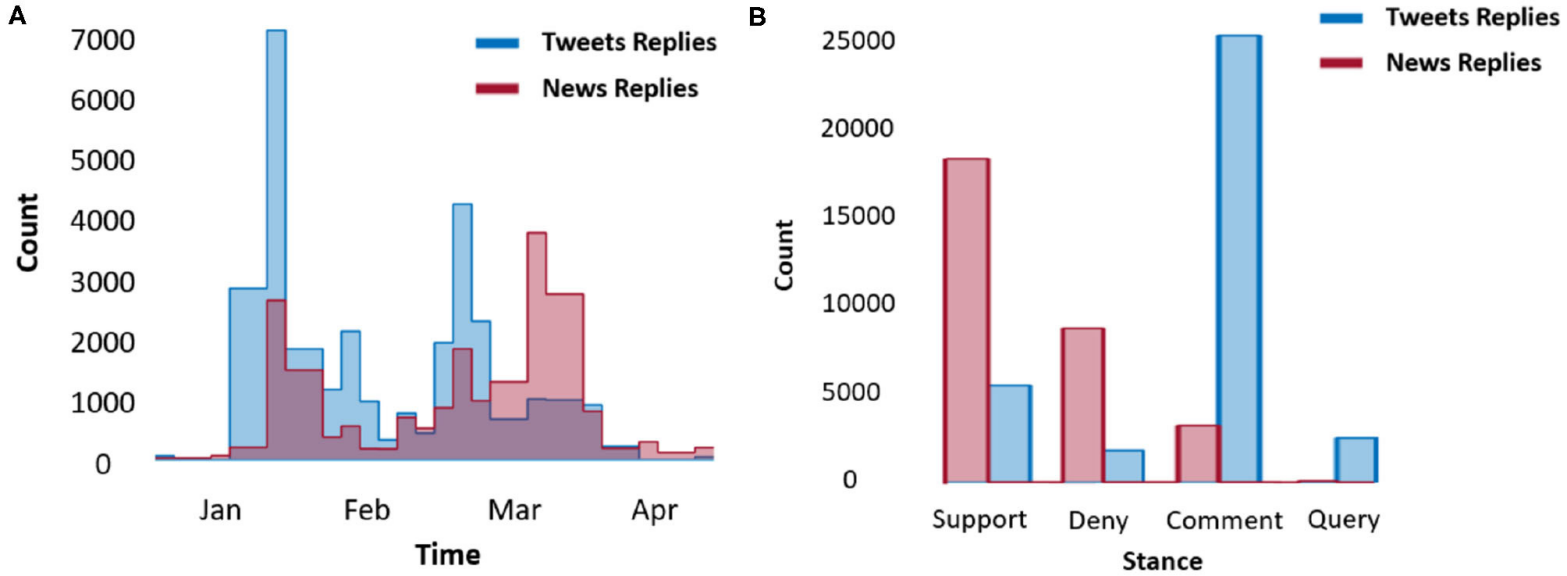
Reply records **(A)** and their stance statistics **(B)** in the collected COVID-19 dataset.

The news dataset contains rumors from news reports about COVID-19, including emergency events, comments of public figures, updates on the coronavirus outbreak, etc. Each record contains the following formatted metadata describing the details of the news:

- **Sources:** Websites containing the rumor sentence. Note that web pages discussing the rumor, e.g., discussing the veracity of the rumor, are counted as sources, and the earliest rumor source is noted as the origin.
- **Popularity:** The popularity of each news is the number of all websites in Google browser that repost the whole rumor.
- **Date:** The publish date of each rumor record, which is collected by the web crawler automatically.
- **Stance:** The attitude of the author or editor of the rumor source. We follow classical rumor stance classification and define four classes of stance: support, deny, comment, and query (Cheng et al., 2020). The stances are labeled and cross-validated manually by going through the context of each website. It is worth noting that the stances are concentrated in support and comment types. Stance statistics of both news and Twitter set are shown in Figure 3.
- **Sentiment:** We define a rumor sentence with one of the five fine-grained types of sentiment: very negative, negative, neutral, positive, and very positive. We manually label and cross-validate the sentiment according to whether it is good news or bad news. For example, news reporting new infections is usually labeled as negative; news indicating death due to COVID-19 would be labeled as very negative; news providing tips of virus prevention is more likely to be labeled as positive; news reporting progress in the scientific research, massive donations, or breakthroughs in the vaccine is rated as very positive.
- **Veracity:** The veracity status of each rumor can be true, representing the news is describing a fact; or false, representing the news is a false claim; or unverified, indicating the news cannot be verified by the time of the collection. The labeling is conducted and cross-validated manually at the data collecting stage based on authoritative websites and shared common knowledge.

### 3.2. Twitter Dataset

The Twitter dataset contains rumors posted on Twitter. The data are collected from public accounts, which comment on the updates of COVID-19-related information discussion under COVID-19-related tags. The discussions may then be retweeted or quoted by other accounts.

**Figure 4:**
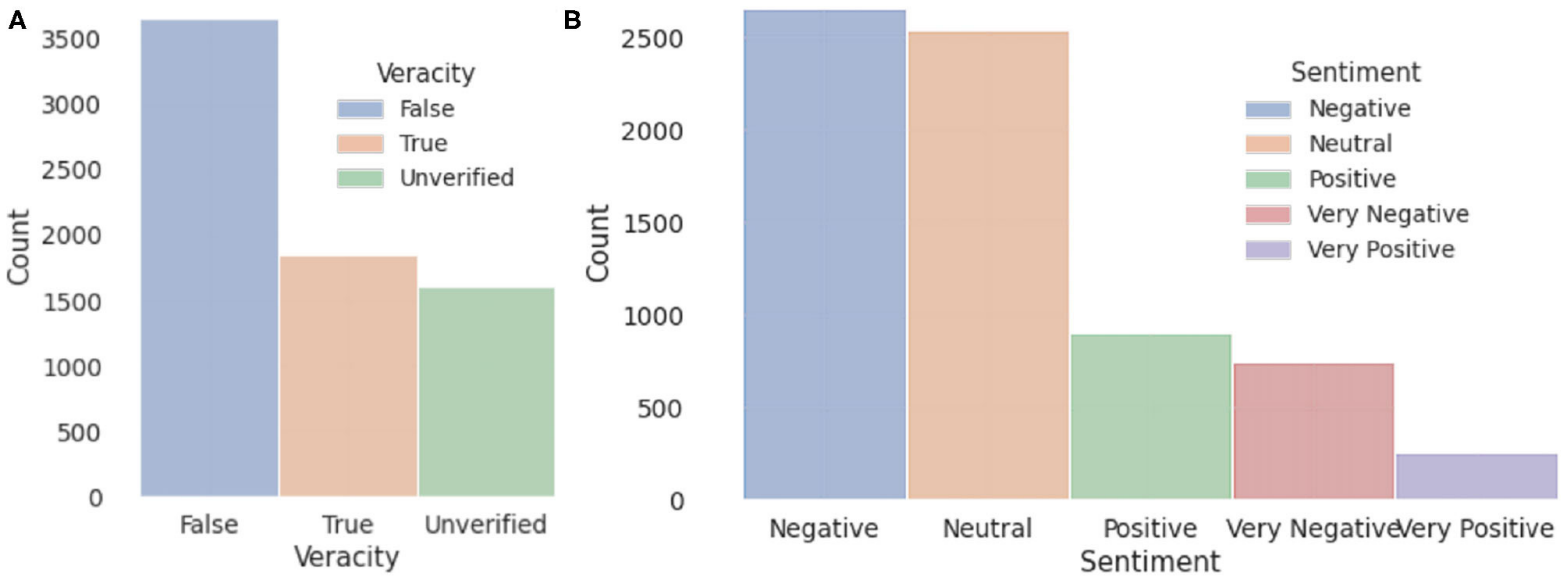
Veracity **(A)** and sentiment **(B)** statistics of our collected dataset.

- **Reply:** Replies indicate how people respond to the tweet, which is similar to sources in news dataset.
- **Reply/Retweet/Like (RRL) number:** These numbers show the propagation trend of a tweet. The RRL number is parsed automatically by the crawler.
- **Popularity:** Popularity of a tweet is represented by the sum of RRL number.
- **Date:** The date is when the tweet is published on the Twitter, which is represented by the format: MM.DD.YYYY.
- **Stance:** Stance represents the attitude of the sources quoting the original tweet or people commenting on it. We provide a stance for rumor itself and its retweets (if any).
- **Sentiment:** We label and cross-validate the sentiment according to the emotion of the tweet. In addition, online tools of sentiment-analysis like MonkeyLearn (MonkeyLearn, 2020) are used for comparisons. We find that compared to our manual assessment, the out-of-box tools make a significant amount of mistakes and lead to low accuracy.
- **Veracity:** The definition is the same as in news dataset. Veracity and sentiment statistics of both Twitter and news sets are shown in Figure 4.

## 4. Technical Validation

We manually label and cross-validate all records by referring to multiple sources. Veracity of a sentence can be true/false/unverified. More specifically, if the data are retrieved from those authoritative fact-checking websites, such as Snopes, Politifact, and Boomlive, we directly use the veracity given by the websites (If a statement appears in multiple sources, we use the voting mechanism and choose the most agreed label). If the data are from social media, we apply the following procedure: (1) Search it on the search engine to see whether any similar case has been given in some authoritative websites. (2) Use common sense to determine, e.g., “drinking bleach can cure coronavirus” is a false statement. (3) If the sentence is not appeared in any formal websites but only on social media, it would be rated as unverified if it does not violate the common sense check.

**Figure 5:**
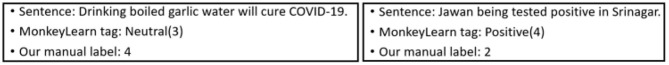
Examples of sentiment comparison between our manual labels and machine-generated labels.

Sentiment label can be one of the five: positive, negative, very positive, very negative, and neutral. The sentiment of tweets are easy to determine, e.g., when including very strong emotion it can either be very positive or very negative. When only mild emotion is expressed, it can either be positive or negative. For rumors collected from news websites, we determine the sentiment by looking at if it includes good/bad news. Specifically, news indicating some new infection is usually labeled as negative; news related to death due to COVID-19 would usually be labeled as very negative; news provides prevention method is usually labeled as positive; while news shows some progress in the scientific research, huge amount of donation, effective government measurement, and/or the progress of the vaccine would be rated as very positive. We note that the above criterion is not absolute; other things like the size of the number in the rumor and the punctuation would also affect its sentiment label. Additionally, 2 out-of-box tools, flair (Akbik et al., 2018) and MonkeyLearn (MonkeyLearn, 2020), are used to cross-validate the outcomes. Flair is a natural-language-processing (NLP) python package from http://github.com/flairNLP/flair, and MonkeyLearn is an online sentiment analysis tool based on natural language processing. Given an input sentence, flair outputs a positive/negative sentiment with a score ranging from 0 to 1. We further evenly divide these ranges into afore-mentioned 5 categories, compare the results with our manual labels, and adjust them accordingly. On the other hand, for each input sentence, MonkeyLearn sentiment classifier picks one tag from the collection (very positive, positive, neutral, negative, very negative), which can be projected directly to the 5 categories defined in section 3. We find that machine-generated labels are not accurate compared to manual labels. 2 typical examples are shown in Figure 5.

Stance label is given to a comment or statement about the misinformation, for example, a reply to a tweet. We label the stance of a sentence as one of the four labels: support, deny, comment, and query. During labeling, we pay attention to semantic features such as “belief,” “report,” “doubt,” and “knowledge.” The support/deny label is given when the sentence clearly supports/denies the veracity of the rumor, e.g., “We doff our hats for your tremendous work. Thank you.” The query label is given when the sentence is raising a question or seeking for additional evidence to confirm the veracity, e.g., “Why do you show this photo?” The comment label is the most frequent out of all labels, and it is given when the sentence does not contain any of the above features. For those comments which have more than one attitude, like support part of the statement but denying others, it would be labeled as the comment.

**Figure 6:**
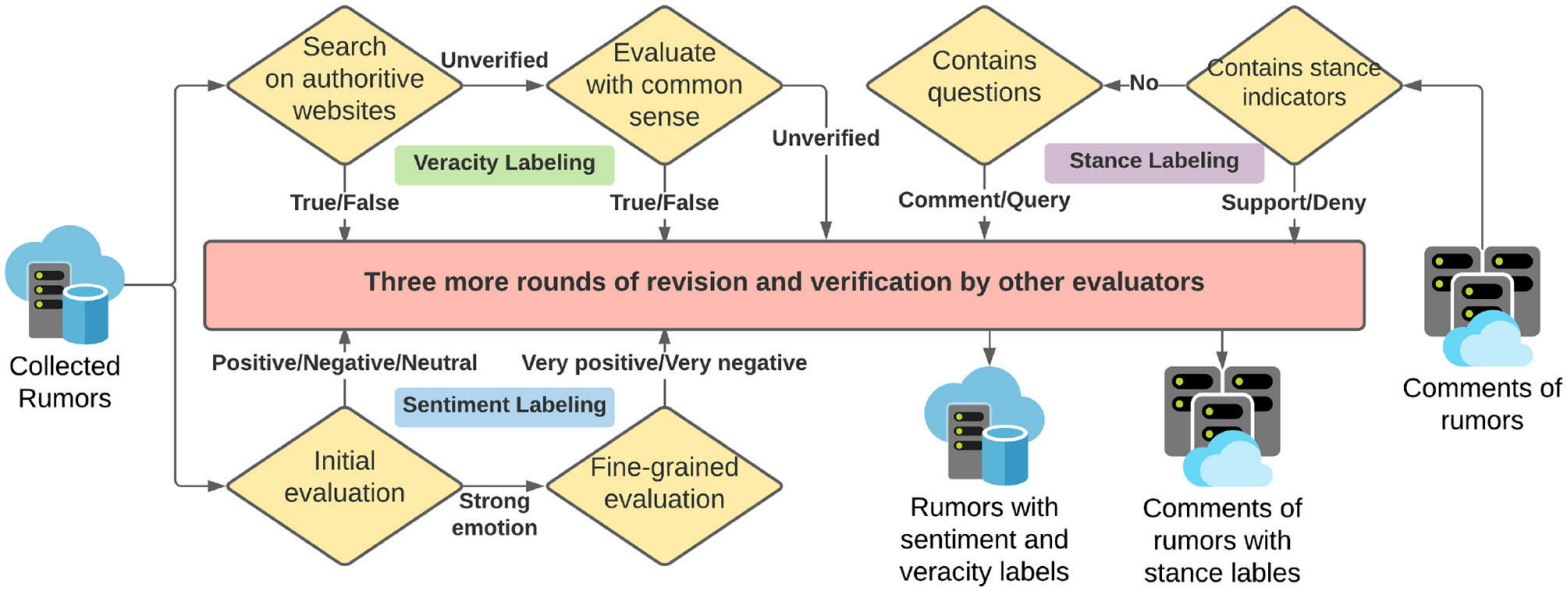
Data labeling pipeline demonstration. We manually label the sentiment, veracity of rumor records, and the stance of comments.

In addition, we went over our labeling more than 3 times for revision and verification. The revision procedure is described as follows. We randomly picked small groups of data as test examples, and our 5 judges give individual judgments, and we hold discussions to compare and discuss our labeling standard, until we reach consistent sense for each example. After this training, we started over again to revise the labels. We provide a logic diagram of our labeling process in Figure 6 and we post our dataset on Github to be publicly available, and we also encourage the readers and users to participate in the type of evaluation. We will keep revising the labels upon and discussion.

## 5. Usage Notes

Our dataset contains COVID-19 rumors and their metadata, such as veracity, sentiment, popularity, etc. In this section, we point out potential data analysis directions and present rumor classification tasks that could be done using our dataset as follows:

- **Rumor analysis**. We analyze the statistics of the rumor data along with the detrended fluctuation analysis of both Twitter and news data.
- **Rumor classification**. We provide a case study of rumor classification tasks with our collected dataset including veracity classification, stance classification, and sentiment analysis.

### 5.1. Top Words and Trending Hashtags in General Tweets Have Different Veracity Patterns

**Figure 7:**
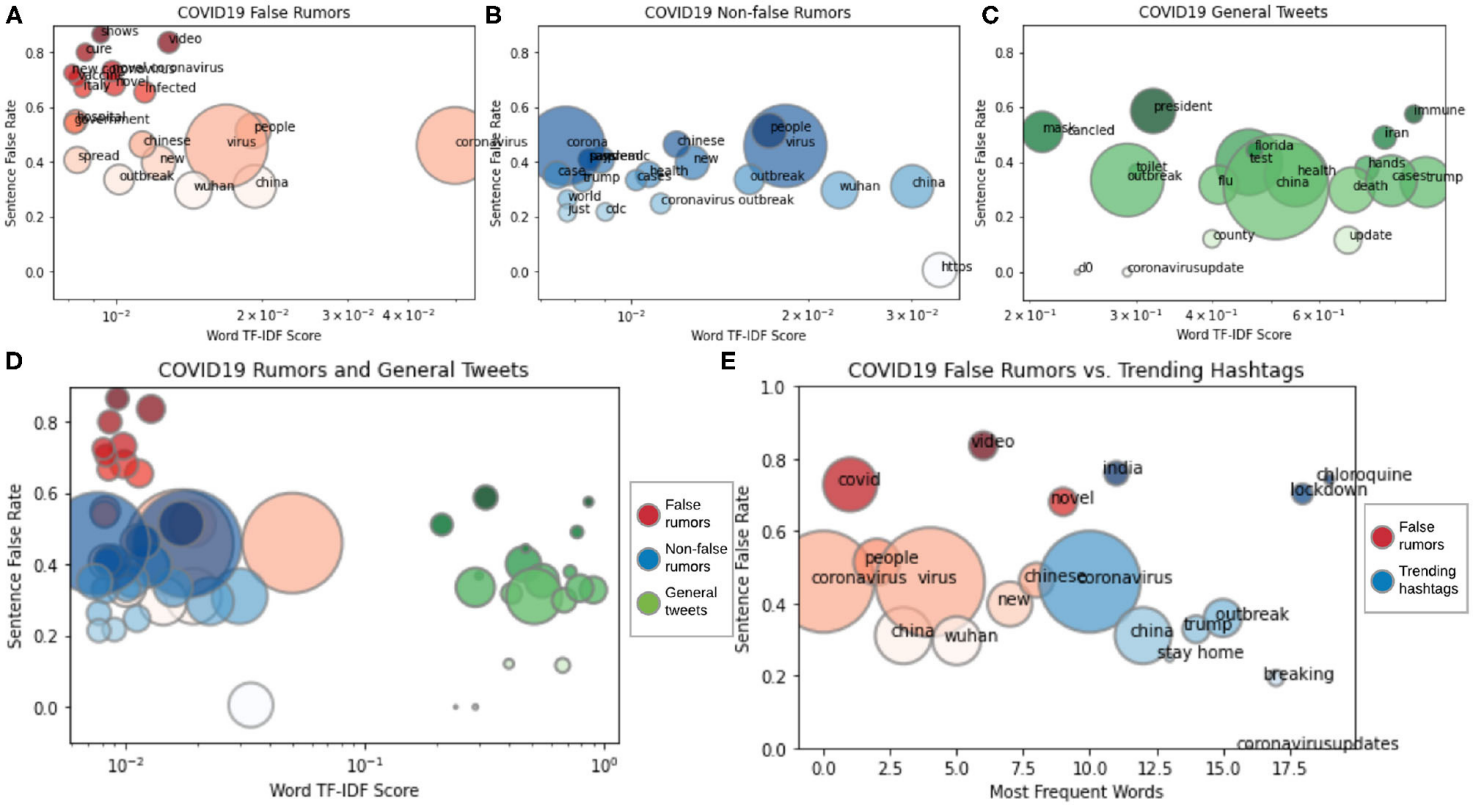
TF-IDF vs. sentence false rate. **(A–D)** Comparison between top words in COVID-19 tweets, false rumors, and non-false rumors. **(E)** Comparison between frequent words in false rumors and trending Twitter hashtags. Color and size of the bubble indicate word source and frequency.

We compare the *top words* in our rumor dataset and general COVID-19 tweets. Words with the highest term frequency-inverse document frequency (TF-IDF) scores (Rajaraman and Ullman, 2011) are referred to as *top words*. We find that words in Twitter posts with the highest TF-IDF scores share very similar *sentence false rate* as top words in our non-false rumors (non-false represents true and unverified rumors). *Sentence false rate* of a word in this work is calculated as the ratio of the occurrence of the word in false rumor sentences and occurrence of the word in any rumors (both false and non-false). If the word “wash” appears in false rumors *f* times and appears in non-false rumors *n* times, then the sentence false rate of “wash” is calculated as *f*/(*f* + *n*). A high sentence false rate indicates a word that appears much more frequently in false rumors than in non-false rumors. Figures 7A–D illustrates the comparison between the TF-IDF and sentence false rate of top words in false rumors, non-false rumors, and general COVID-19 tweets. We find that top words in tweets appear almost equally in false rumors and non-false rumors, i.e., the green bubbles in Figure 7C have around 0.5 sentence false rate. Top words in our non-false rumors have the same patterns as top words in general COVID-19 tweets, as shown in Figure 7B, i.e., the blue bubbles have around 0.5 sentence false rate. The sentence false rate similarity between top words in general COVID-19 tweets and our non-false rumors indicates that the general veracity/truthfulness of COVID-19 Twitter posts are toward non-false.

Different from top words in general Twitter posts and non-false rumors, we find that top words in our false rumors (marked as red bubbles) have different patterns, as shown in Figure 7A. The red bubbles lying in a much higher sentence false rate range, [0.4, 1.0], indicate that the top words in false rumors, such as “cure” and “vaccine,” appear much more frequently in false rumors than in non-false rumors. These words are “false rumor-specific,” meaning that other words that are frequently seen in non-false rumors, such as “outbreak” and “cases,” do not share the same significance as “cure” or “vaccine” in false rumors.

After comparing the top words in general COVID-19 tweets and our rumor dataset, we analyze the trending coronavirus-related Twitter hashtags and find that the steady trending hashtags behave similarly to *the most frequent words* in false rumors. *The most frequent words* are those that appear most frequently in some content, in our case, false rumors. As shown in Figure 7E, the trending hashtags in Twitter (marked as blue bubbles), such as “India,” “lockdown,” and “chloroquine,” and most frequent words in false rumors (marked as red bubbles), such as “covid,” “video,” and “novel,” lie in similar sentence false rate range, meaning that the veracity of trending hashtags is at the same level as false rumors.

### 5.2. Detrended Fluctuation Analysis

We use the DFA (Peng et al., 1994; Ihlen, 2012) (Detrended Fluctuation Analysis) to analyze the long-range correlation of the collected time series. DFA is a scaling analysis method to analyze the time series (rumor popularity) that appear to possess long-range memory properties. The Hurst exponent (Lloyd, 1966) (α) characterized by the power–law relationship between the root mean square fluctuations and the window scales represents the long-range correlation properties of the time series. When the Hurst exponent α > 0.5, it indicates that the time series has obvious long-range memory properties; the higher the Hurst exponent α is, the stronger the higher order correlation structure.

**Figure 8:**
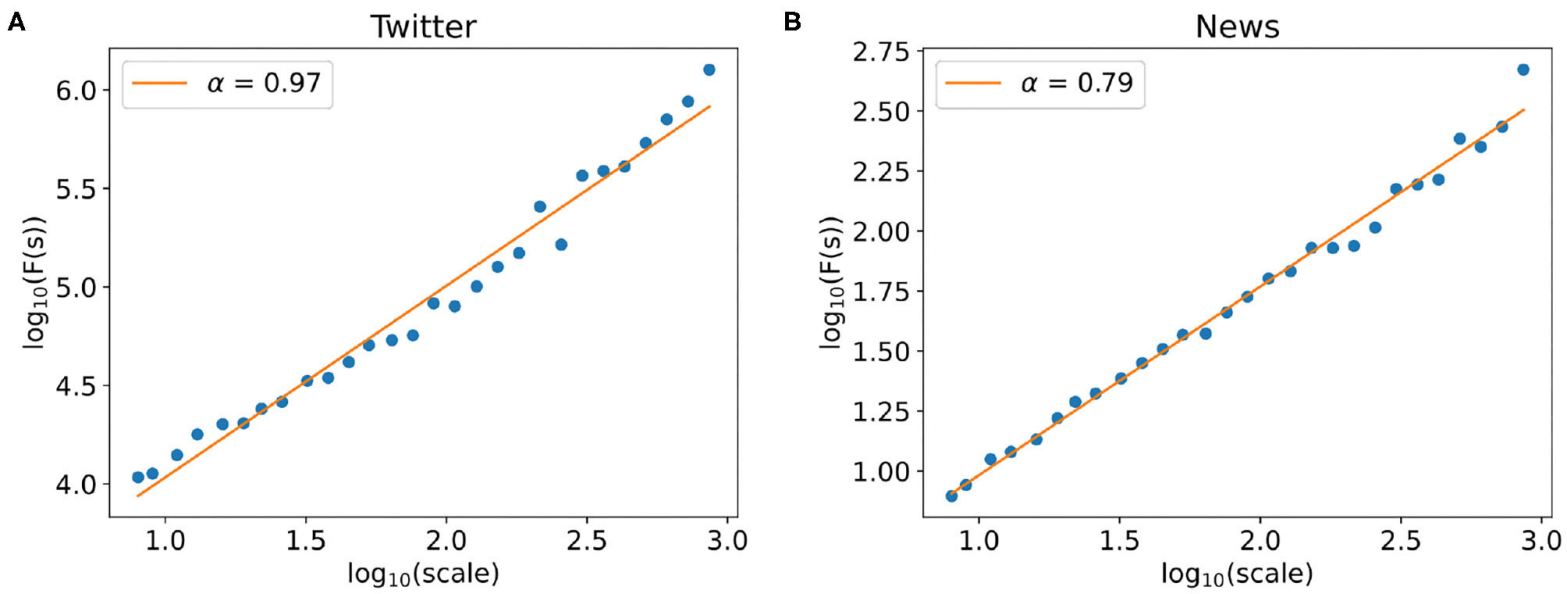
Detrended fluctuation analysis of time series. **(A,B)** The power–law relationship between the fluctuation F(s) and window scale of the Twitter popularity time series **(A)** and the News popularity time series **(B)**.

Here, we apply the DFA method to the Twitter popularity time series and the News popularity time series. The power–law correlation between the fluctuation function *F*(*s*) and window scale *s* is shown in Figure 8, in which we learn the Hurst exponent of the Twitter series is α_*Twitter*_ = 0.97, while the Hurst exponent of News series is α_*News*_ = 0.79. It indicates that both the Twitter series and the News series have obvious long-range correlations, showing that the variation of the time series is not a random Markovian process. The characteristic of the past series will affect the statistics of the current and future series, that is, there is a memory effect in the time series. Meanwhile, the Twitter time series has a higher Hurst exponent, which means that there is a stronger positive correlation between the historical variation trends and the future fluctuation trends.

### 5.3. Deep Learning-Based Rumor Classification Tasks

**Figure 9:**
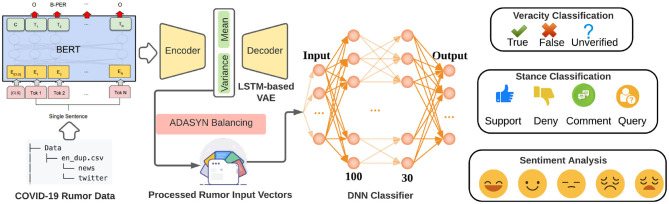
Our rumor deep learning-based classification system. Textural sentences in our dataset are first converted to vectors using Bidirectional Encoder Representations from Transformers (BERT), and then sent through an LSTM-based Variational AutoEncoder (VAE) to extract features. After the ADASYN balancing, the processed rumor input vectors are taken by the DNN classifier as input. Three classification tasks (veracity, stance, and sentiment) are finally performed separately.

We implemented a BERT (Devlin et al., 2018) and VAE (Kingma and Welling, 2013)-based rumor classification system in this section as shown in Figure 9. Our classification system is applied to 3 tasks: rumor veracity classification, stance classification, and sentiment classification. The statistics of the veracity, sentiment, and stance in our dataset are shown in Figures 3, 4. In our experiment, BERT is fine-tuned to serve as a word embedding layer that converts rumor textural input to vectors. The BERT is a representation model pre-trained by jointly conditioning on both the left and right context in all layers. Therefore, deep bidirectional representations of unlabeled text on both left and right contexts in all layers can be learned. In the next step, we follow VRoC (Cheng et al., 2020) and use an LSTM-based VAE to extract features from the vectors generated by BERT. Then, we use ADASYN (He et al., 2008) to balance the data. Finally, we utilize a 4-layer fully connected neural network to perform classification. Note that we use the same classifier architecture in all tasks and a five-fold cross-validation is performed.

**Figure 10:**
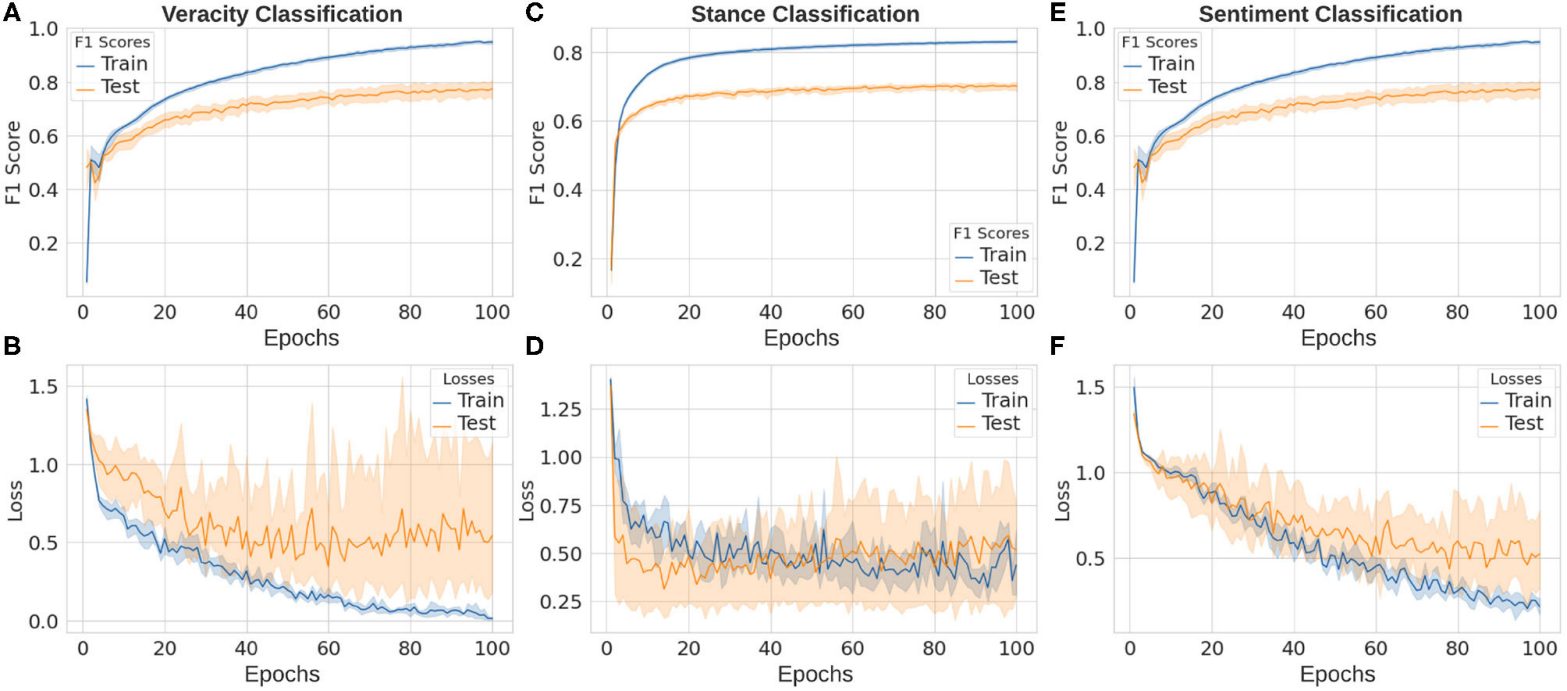
Deep learning results in our rumor classification tasks: veracity classification **(A,B)**, stance classification **(C,D)**, and sentiment classification **(E,F)**.

The false rumor tends to contain certain patterns from which some lexical and syntactic features can be extracted to distinguish a true statement from a fake one. Veracity classification or false rumor detection plays an important role in rumor classification systems (Cheng et al., 2021a). In our deep learning based veracity classification task, we take our collected rumor data and use VAE as a feature extractor to learn representations from input vectors. The extracted features then are utilized to classify true, false, and unverified rumors. The final macro F1 score of our veracity classification task is 85.98 ± 0.10% and the performance of our classifier is shown in Figures 10A,B.

The classification of stances is obviously a harder task for two reasons: (i) stance in our dataset has more categories that makes the classification naturally more difficult; (ii) it is not parts, but the complicated semantic meaning of the whole commenting sentence (and even original sentence being commented on) that should be learned to understand its stance. In our experiments, with the same deep learning architecture, the performance on stance classification task is lower than that of veracity task, which is 70.25 ± 0.02% as shown in Figures 10C,D.

Sentiment analysis is also a vital task in natural language processing, text classification, and rumor-related tasks. Similar to the case in stance classification, classifying emotions within a sentence is also very tough because of the high complexity of comprehending the sentence and its context. The final macro F1 score of our deep learning architecture on the sentiment classification is 77.32 ± 0.16% as shown in Figures 10E,F.

## 6. Discussion

In this work, we collect a COVID-19 rumor dataset including rumors from Twitter and news websites, and we provide the meta data of rumors such as reply/retweet/likes number, comments, publish date, veracity labels, sentiment labels, and stance labels. We envision the downstream applications or usage cases of this dataset to include but not restricted to (i) the identification, prediction, classification of rumor, misinformation, disinformation, and fake news; (ii) the study of rumor spread trend and rumor/misinformation/disinformation/fake news combating and/or control; (iii) social network and complex network-related studies in terms of information flow and transition; and (iv) the natural language processing-related studies of rumor sentiment and semantic. Since for information diffusion, we only considered first-order reply, retweet, and likes, the second and higher order diffusion are not collected due to Twitter policies, one limitation of this dataset lies in the information diffusion capture and related downstream applications. In our future work, we will study the data collected in depth and working on higher order meta data collection.

## Data Availability Statement

The datasets presented in this study can be found in online repositories. The names of the repository/repositories and accession number(s) can be found below: https://github.com/MickeysClubhouse/COVID-19-rumor-dataset.

## Author Contributions

MC, SN, and PB contributed to the design of the research including data collection, data processing, data analysis, experiments, and organizing the information in the main text. MC, SW, TY, XY, ZH, SN, and PB contributed to the writing of the manuscript. SW, TY, XY, and WW contributed to the data collection and labeling. WW contributed to the crawler implementation. MC, XX, and ZH contributed to the implementation of data analysis experiments. MC and XY contributed to preparing the figures and tables, and their captions. All authors reviewed the manuscript.

## Conflict of Interest

The authors declare that the research was conducted in the absence of any commercial or financial relationships that could be construed as a potential conflict of interest.
